# In vitro safety evaluation of rare earth-lean alloys for permanent magnets manufacturing

**DOI:** 10.1038/s41598-021-91890-0

**Published:** 2021-06-16

**Authors:** Carlos Rumbo, Cristina Cancho Espina, Jürgen Gassmann, Olivier Tosoni, Rocío Barros García, Sonia Martel Martín, Juan Antonio Tamayo-Ramos

**Affiliations:** 1 International Research Center in Critical Raw Materials-ICCRAM, Universidad de Burgos, Plaza Misael Bañuelos s/n, 09001 Burgos, Spain; 2 Fraunhofer Research Institution for Materials Recycling and Resource Strategies IWKS, Aschaffenburger Straße 121, 63457 Hanau, Germany; 3 CEA-LITEN, 38054 Grenoble, France

**Keywords:** Environmental impact, Microbiology, Cell biology

## Abstract

Due to their exceptional physico-chemical and magnetic characteristics, rare earth (RE) permanent magnets are applied in multiple critical technologies. However, several environmental and economic difficulties arising from obtaining RE elements have prompted the search of alternatives with acceptable magnetic properties but containing a lower percentage of these elements in their composition. The aim of this work was to perform a preliminary toxicological evaluation of three forms of newly developed RE-lean alloys (one NdFeTi and two NdFeSi alloys) applying different in vitro assays, using as a benchmark a commercial NdFeB alloy. Thus, the effects of the direct exposure to powder suspensions and to their derived leachates were analysed in two model organisms (the A549 human cell line and the yeast *Saccharomyces cerevisiae*) applying both viability and oxidative stress assays. Moreover, the impact of the alloy leachates on the bioluminescence of *Vibrio fischeri* was also investigated. The obtained data showed that only the direct interaction of the alloys particulates with the applied organisms resulted in harmful effects, having all the alloys a comparable toxicological potential to that presented by the reference material in the conditions tested. Altogether, this study provides new insights about the safety of NdFeTi and NdFeSi alloys.

## Introduction

Rare earth (RE) elements have a key role in many high technologies, including the development of permanent magnets^1^, where their exceptional physico-chemical characteristics and their magnetic properties make them excellent candidates to be used in the manufacturing of these materials. Among them, neodymium (Nd) stands out as one of the preferred elements in the permanent magnet fabrication, being the neodymium–iron–boron (NdFeB) the strongest commercially available permanent magnet. Developed in the 1980s, and belonging to the third generation of permanent magnets^2^, NdFeB magnets display extraordinary properties^3^ for their application in several fields, such as the automotive sector^4,5^, dentistry^6^ or electronic products^7^.

Due to several economic and political factors^8^, as well as to the insufficient availability of RE elements, Nd permanent magnets are commercially expensive. In addition, their high demand, which is expected to increase due to the dependence on permanent magnets of the electrical vehicle motors, will result in a rise in their price, as observed in the last years^9^. This uncertainty in the market of RE elements, combined with the environmental implications of their extraction^10^, such as the accumulation of radioactive tailings due to the radioisotopes contained in the RE elements deposits^11^, have prompted efforts to develop strategies to reduce the needs of these elements in the permanent magnet field, becoming a priority worldwide. The development of RE-lean permanent magnets with a significant reduction in the percentage of these elements in their composition is one of the strategies that can be followed to accomplish this aim. Within this approach, RE(Fe,M)_12_ compounds, where M represents the transition metals, are considered interesting options for their high magneto crystalline anisotropy, high saturation magnetization and lower RE content^12^. Hence, NdFeSi and NdFeTi, two types of the alloys belonging to this group^13,14^, show excellent magnetic properties that make them good candidates for their implementation in the fabrication of permanent magnets with a reduced RE percentage in their composition.

Since metals have an important role in a broad range of biological processes, these compounds are essential to living systems^15^. Consequently, homeostasis of metal ions is critical, and their levels need to be kept within strict limit^16^, outside of which their lack or excess can lead to adverse effects. The toxicological impact of metal compounds is an issue that has been widely studied, existing numerous research works where the general aspects and the mechanisms involving their toxicity were reviewed^17,18^. In general, organisms are exposed to metal mixtures in the environment but, in most cases, toxicological studies are performed considering the effects of the metals independently. In this regards, some research works showed that the effects of metal combinations can pose a higher toxicological impact than these materials individually^19^, constituting a relevant factor that should be taken into account when the potential risks associated to metals are being assessed. Among the different pernicious effects that these compounds may cause, oxidative stress is a common feature of metal toxicity, where the generation of reactive radicals can produce different harmful effects such as DNA damage, lipid peroxidation or protein depletion^20^.

Despite the wide application of RE-permanent magnets in several fields, the information about their safety is scarce. The implementation of NdFeB magnets in dentistry led to the emergence of a few studies where the cytotoxicity of pieces of this material and their associated corrosion products were addressed^21–23^. However, the results observed were in some cases conflicting, which confirms the need to develop more toxicological research on Nd magnets. Regarding the potential hazards of the alloys applied in the fabrication of permanent magnets in powder form, to our knowledge, currently there is only one work performing a preliminary toxicological evaluation of these materials and their associated leachates^24^. Thus, due to this lack of studies, the provision of data about the possible risks of the metal powders of RE-magnets, as well as of those of new alternatives for the current commercial magnets is crucial, since it will provide relevant information about their potential effects before their application.

Considering that NdFeTi and NdFeSi are magnetic alloys intended for substituting current permanent NdFeB magnets, the aim of this work was to evaluate the potential toxicity of three new developed types of these alloys (two types with the composition (Nd,Zr,Y)Fe_10_Si_2_ and one type with the composition (Nd,Pr)_1.15_Fe_11_Ti) in powder form, and their associated leachates. For that purpose, both viability and oxidative stress assays were performed using the human cell line A549 and the yeast *Saccharomyces cerevisiae*. In addition, the safety of the alloys leachates was also assessed through the bioluminescent inhibition assay using the Gram negative bacterium *Vibrio fischeri*. A commercial Nd_2_Fe_14_B powder alloy was used in the assays to establish reference values in the studied conditions. Altogether, the presented data provide a preliminary evaluation of the safety of NdFeSi and NdFeTi alloys.

## Results

### Characterization of the alloys and their associated leachates

The surface elemental composition of the three RE-lean alloys and the reference sample was semi-quantitatively analysed through SEM–EDX. Table 1 shows the values obtained for each of the main elements comprising the alloys (wt%), with the exception of B, which cannot be detected through EDX as it presents low photon energy. As expected, the reference sample showed the highest percentage of Nd in its composition (≈ 16 wt%), while both NdFeSi samples showed the lowest content of this element (≈ 5 wt%). In the NdFeTi sample, the reduction of Nd was not as evident as in the other RE-lean alloys, presenting a 13.78 wt% of this element in its composition. However, it can be considered as a relevant reduction, since it represents a decrease of ≈13% with respect to the Nd content in the reference. By the same token, relevant amounts of other elements that were added during the alloys production as a replacement for some of the Nd were also quantified. Thus, Pr was detected in the NdFeTi sample (4.27 wt% ± 0.39) while significant percentages of Zr (9.29 wt% ± 2.82 and 8.89 wt% ± 0.80) were found in both NdFeSi S1 and NdFeSi S2 samples respectively. Y, which was also employed for the fabrication of NdFeSi alloys, was not detected in any of the samples.

**Table 1:** Average weight percentage (wt%) of elements in the selected alloys determined by SEM–EDX.

		NdFeB
Nd		15.97 ± 2.84
Fe		62.93 ± 3.43
B		–

	NdFeSi S1	NdFeSi S2
Nd	5.09 ± 0.51	4.97 ± 0.35
Fe	73.52 ± 5.69	73.10 ± 2.48
Si	6.51 ± 1.40	6.92 ± 0.48

		NdFeTi
Nd		13.78 ± 0.68
Fe		69.96 ± 1.19
Ti		5.72 ± 0.23

**Figure 1:**
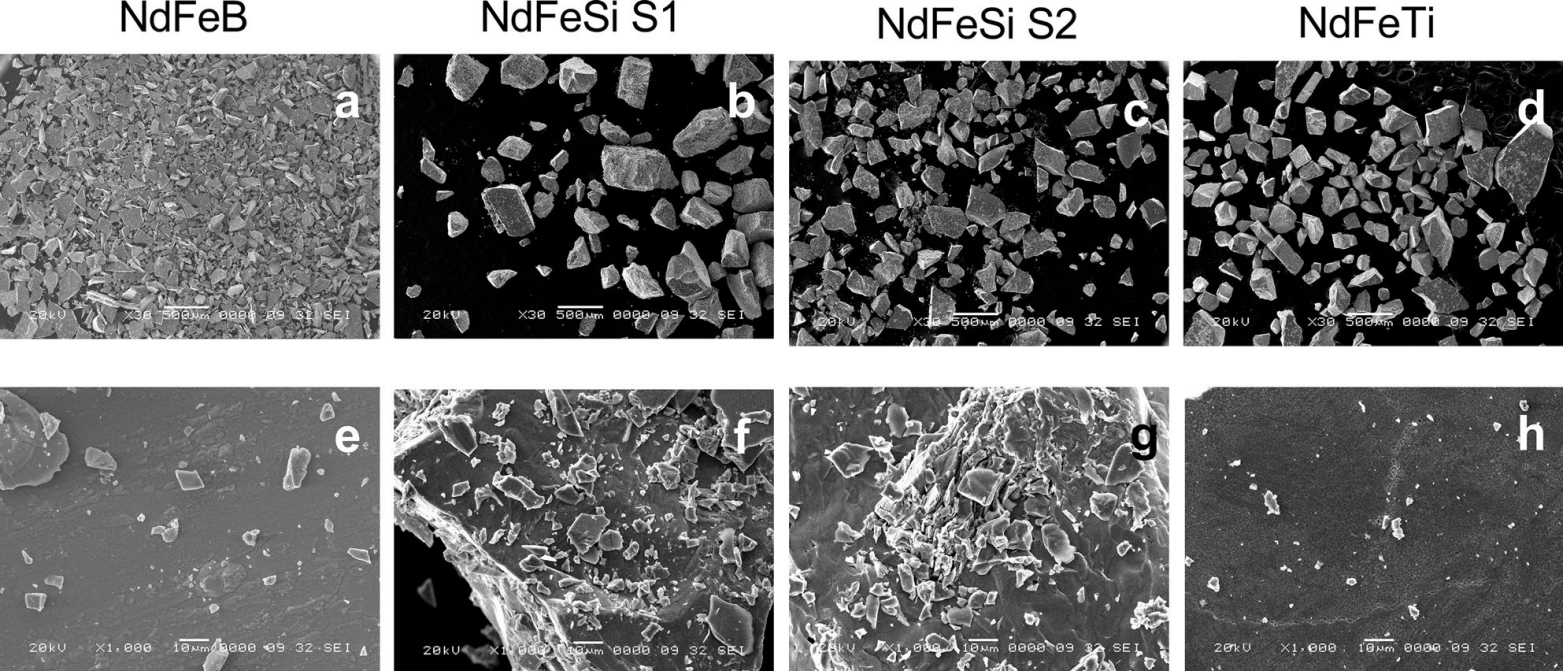
SEM images showing the morphology of the magnet powders. NdFeB (**a**,**e**); NdFeSi S1 (**b**,**f**); NdFeSi S2 (**c**,**g**) and NdFeTi (**d**,**g**). Images a, b, c and d: Original magnification × 30 (Scale bar = 500 μm); Images e, f, g and h: Original magnification × 1000 (Scale bar = 10 μm).

The morphology and the size of the particles that form the alloy powders were also analysed and visualized by SEM. For this purpose, a small amount from each sample was directly used for the observations. Figure 1 shows the appearance of the particles in NdFeB (1a, 1e), in both NdFeSi (1b and 1f; 1c and 1 g) and in NdFeTi samples (1d, 1 h). All of them showed to be formed of a heterogeneity of particles that present a great variety of dimensions that range from a few to several hundred micrometers. The biggest particles were observed in the RE-lean alloy powders, where some of them presented sizes above 500 µm, while in the commercial NdFeB powders the particles were slightly shorter than the others, without exceeding the 500 µM. Regarding their morphology, a variety of shapes was distinguished, appearing polygonal and round particles. Moreover, in some of the images (Fig. 1e–h), particles in the nanoscale range were observed.

ICP-MS analysis was performed on the alloy leachates to quantify the concentrations of the different dissolved metal ions released from the powders at 10 g/L in water after a 3-month incubation. The concentrations of Nd, Fe and Si were analysed in NdFeSi S1 and NdFeSi S2 leachates. The obtained results showed that the concentration of Nd was higher in NdFeSi S1 than in NdFeSi S2 (988.14 ppb and 651.38 ppb respectively). Regarding Fe and Si, these ions were found in higher concentrations in the leachates from NdFeSi S2, where the obtained values were 9.06 and 234.51 ppb for Fe and Si, whereas in NdFeSi S1 the concentrations observed were 1.67 and 132.01 ppb respectively. In the case of the leachates from NdFeTi, Nd and Fe were also detected (1602.59 and 1.43 ppb respectively) while the levels of Ti were under the limit of detection (< 0.072 ppb). Finally, the levels of Nd, Fe and B were also quantified in the reference sample, presenting values of 0.68, 1.51 and 583.94 ppb. Moreover, ions belonging to the other elements added to reduce the amount of Nd required for the alloy fabrication were also detected. Thus, Pr was quantified in the NdFeTi leachates (1153.60 ppb). For its part, Y was found in the leachates obtained from NdFeSi samples (55.16 and 442.39 ppb in NdFeSi S1 and NdFeSi S2 respectively). On the other hand, the concentrations of Zr, which was also used for the fabrication of both NdFeSi samples, were under the detection limit in both samples (< 0.005 ppb).

### Toxicity studies using A549 cell line

**Figure 2:**
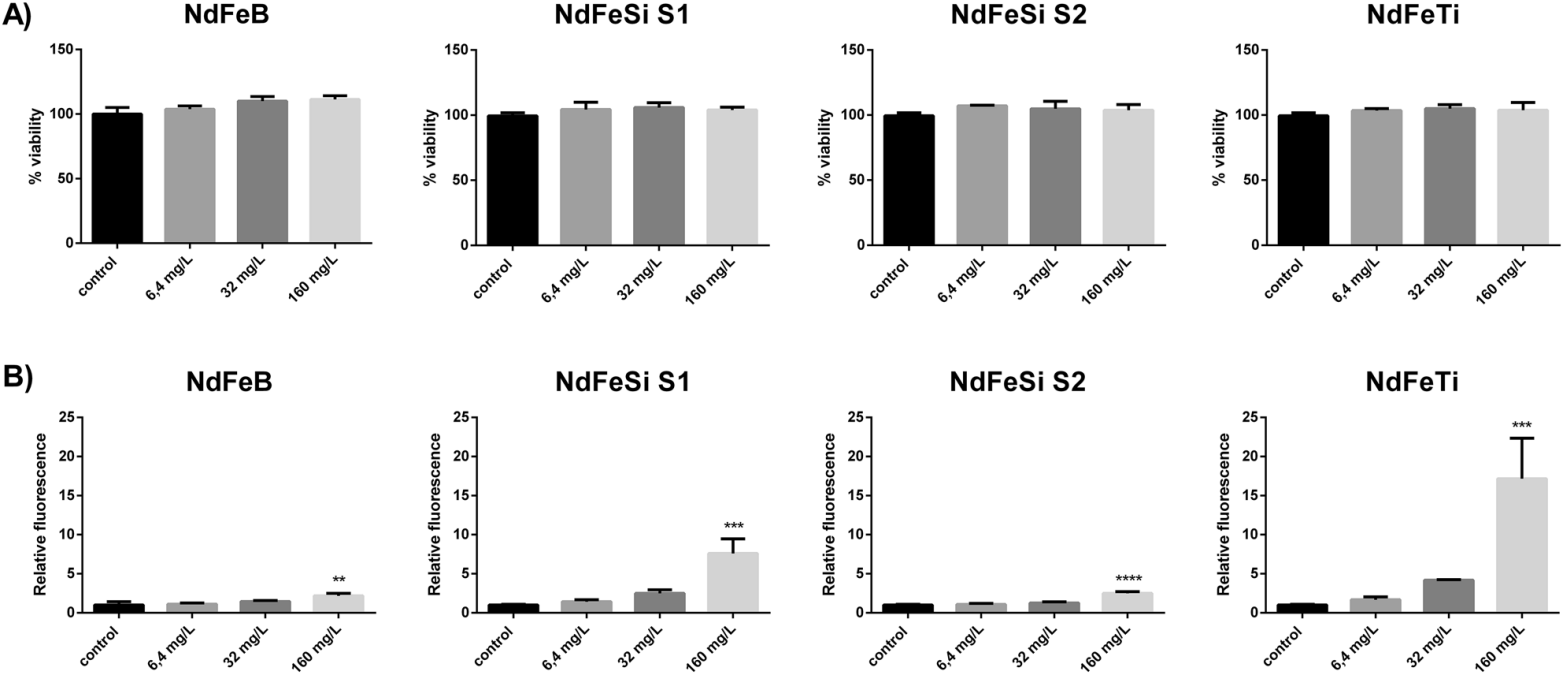
Effects of direct exposition of A549 cells to different concentrations of the alloys suspensions. (**A**) Viability of A549 cells (Neutral Red assay). Results are expressed as % of control (untreated cells). (**B**) Oxidative stress (ROS levels) in A549 cells. Results are expressed as the relative fluorescence value to the control (untreated cells) which was assigned a value of 1. Data represent the mean of 3 biological replicates (± standard deviation, SD). Differences were established using a One-way ANOVA followed by Dunnett post hoc test to compare every mean with the control, and considered significant at *P* ≤ 0.05. **P ≤ 0.01, ***P ≤ 0.001, ****P ≤ 0.0001.

The neutral red uptake assay was applied to study the impact on cell viability after a direct exposure of the A549 cells to different concentrations of the alloys. This assay is based on the ability of the neutral red dye to be uptaken by cells and stain the lysosomes in viable cells, which can be detected and measured. Figure 2A displays the results obtained after exposing cells to 6.4, 32 and 160 mg/L of the different alloys. Results showed that cell viability was not affected after 24 h of incubation with any of the samples. On the other hand, A549 cells directly exposed to the alloys suspensions showed to suffer oxidative stress when they were incubated for one hour at the highest concentrations tested. As shown in Fig. 2B, the levels of reactive oxygen species (ROS) were statistically significant in cells after being exposed to 160 mg/L of all the alloys. Both NdFeB and NdFeSi S2 samples displayed similar levels of ROS at that concentration, which showed to be slightly increased compared to the control condition. For its part, this induction was much higher in NdFeSi S1 and NdFeTi samples, where the levels of ROS were more than 3 and 8 times respectively than those produced by the others at the same conditions (Fig. 2B).

**Figure 3:**
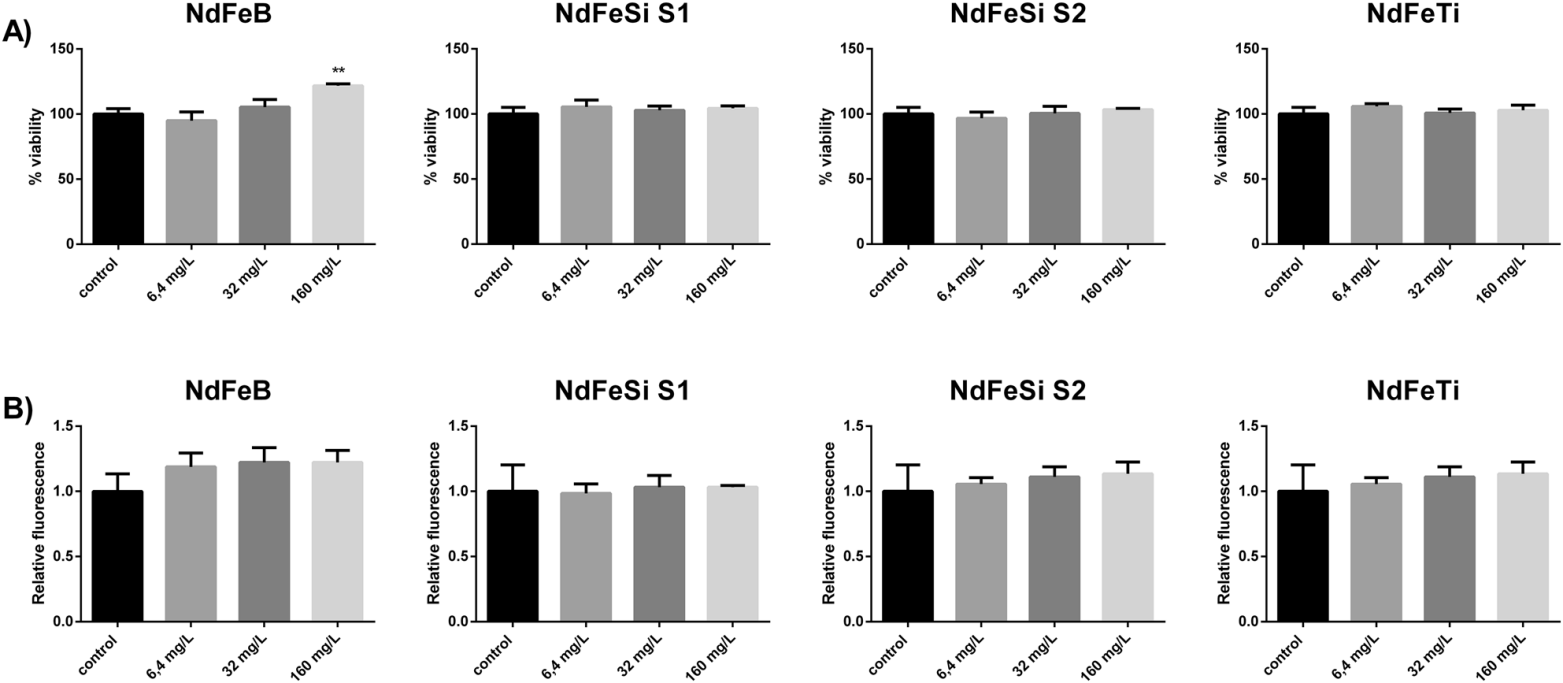
Exposition of A549 cells to different concentrations of the alloy leachates. (**A**) Viability of A549 cells (Neutral Red assay). Results are expressed as % of control (untreated cells). (**B**) Oxidative stress (ROS levels) in A549 cells. Results are expressed as the relative fluorescence value to the control (untreated cells) which was assigned a value of 1. Data represent the mean of 3 biological replicates (± standard deviation, SD). Differences were established using a One-way ANOVA followed by Dunnett post hoc test to compare every mean with the control, and considered significant at *P* ≤ 0.05. **P ≤ 0.01.

Regarding the assays performed using the leachates, cells were exposed to dilutions equivalent to concentrations of 6.4, 32 and 160 mg/L of each alloy. None of them showed to have any significant toxic effect on neither cell viability nor oxidative stress in the conditions tested (Fig. 3).

### Toxicity studies using *Saccharomyces cerevisiae*

**Figure 4:**
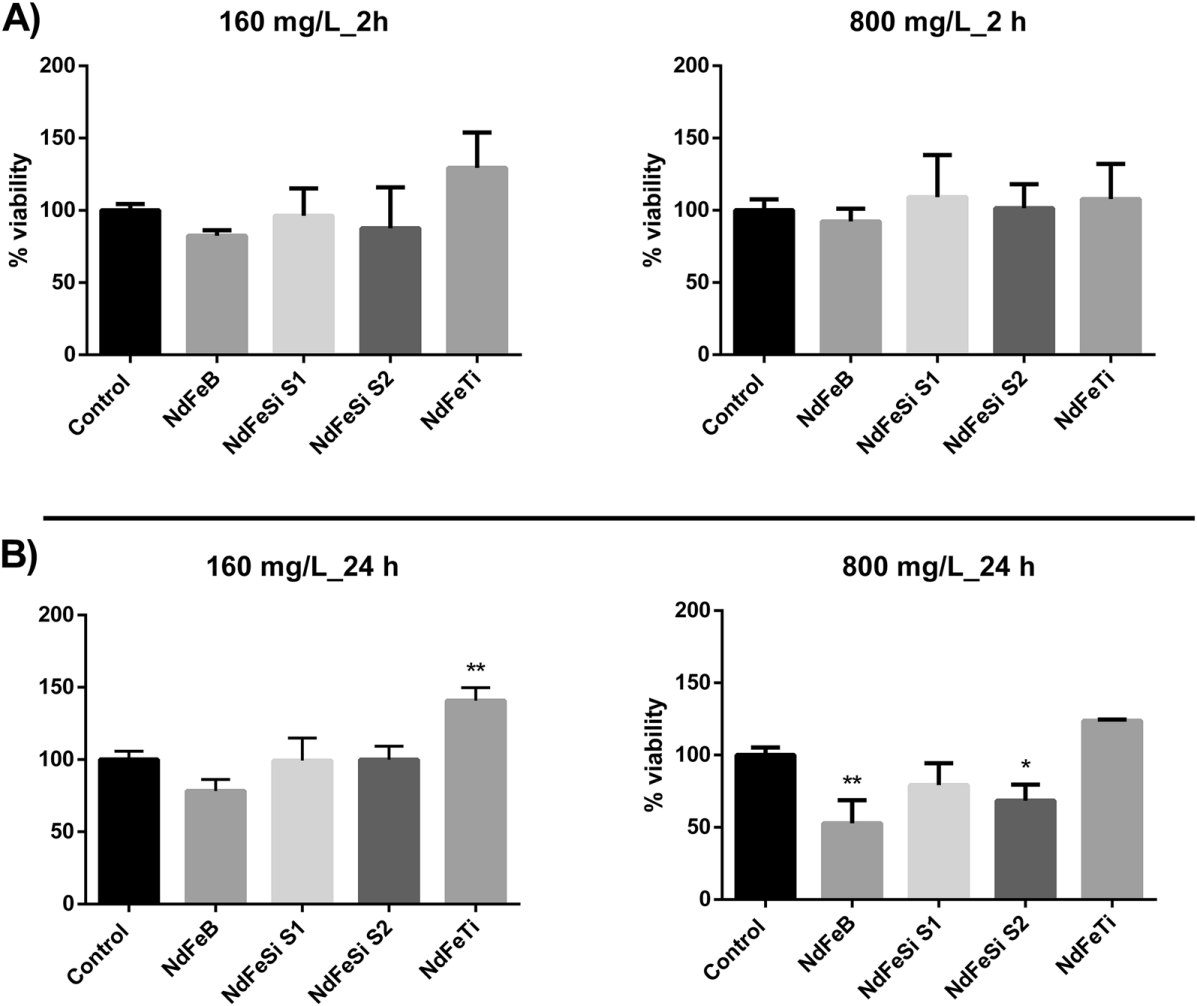
Colony forming units (CFUs) of *S. cerevisiae* cells exposed to different alloys suspensions at two concentrations (160 and 800 mg/L) and two exposure times: 2 h (**A**) and 24 h (**B**). Results are expressed as the percentage (%) of CFUs determined for each exposure condition using as reference value the non-exposed cells condition, which was assigned a value of 100%. Data represent the mean of 3 biological replicates (± standard deviation, SD). Differences were established using a One-way ANOVA followed by Dunnett post hoc test to compare every mean with the control, and considered significant at *P* ≤ 0.05. *P ≤ 0.05, **P ≤ 0.01.

Due to the mechanisms behind the response to metal toxicity are highly conserved between humans and yeasts, *S. cerevisiae* has been extensively applied to elucidate how metals and metalloids affect both biological systems^25,26^. To determine the toxicological potential of the synthesized alloys, this fungal model was included in the assays. Two concentrations (160 and 800 mg/L) and exposure times (2 and 24 h) were assessed (Fig. 4). At the shorter exposure time, no significant differences in the yeast viability were observed in any of the studied conditions (Fig. 4A). However, after a longer exposure period (24 h), the decrease of yeast CFUs in some cases indicated an impact of the alloy suspensions on the cell´s viability (Fig. 4B). In the presence of 160 mg/L, no negative effects on the viability of *S. cerevisiae* cells could be detected. However, in the presence of 800 mg/L, a decrease in the average cells viability was observed in cells exposed to NdFeB, NdFeSi S1 and NdFeSi S2 samples, being only statistically significant in the latter sample and in the case of the reference alloy.

**Figure 5:**
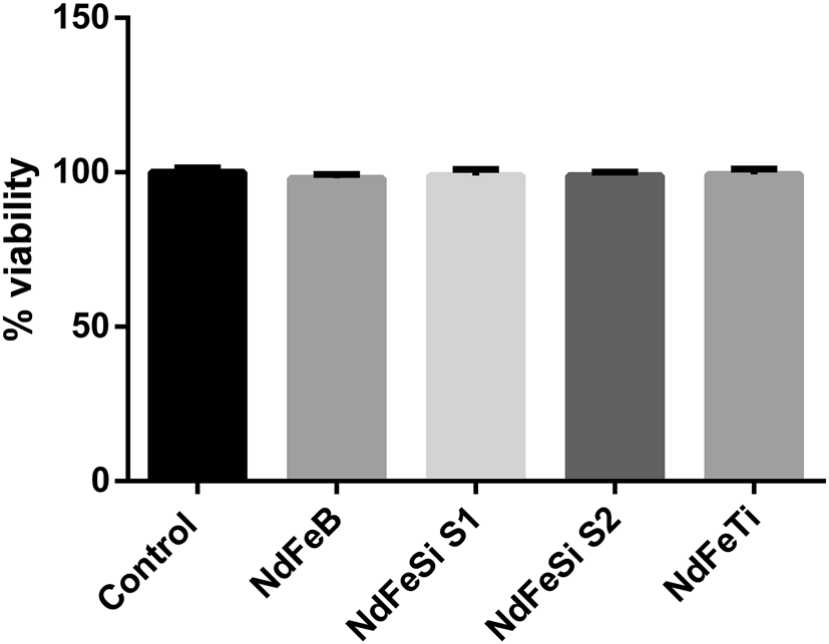
CFUs of *S. cerevisiae* cells exposed to different alloys leachates during 24 h. Results are expressed as the percentage (%) of CFUs determined for each exposure condition using as reference value the non-exposed cells condition, which was assigned a value of 100%. Data represent the mean of 3 biological replicates (± standard deviation, SD). Differences were established using a One-way ANOVA followed by Dunnett post hoc test to compare every mean with the control condition, and considered significant at *P* ≤ 0.05.

The toxicological potential of the alloys leachates was also evaluated on *S. cerevisiae*, using the same exposure conditions. Yeast cells were exposed to a leachate equivalent to a concentration of 800 mg/L of each alloy for 24 h (Fig. 5). Differently to what was observed in case of the direct contact experiments, none of the leachate samples produced a negative impact in the viability of this organism in the studied conditions (Fig. 5).

**Figure 6:**
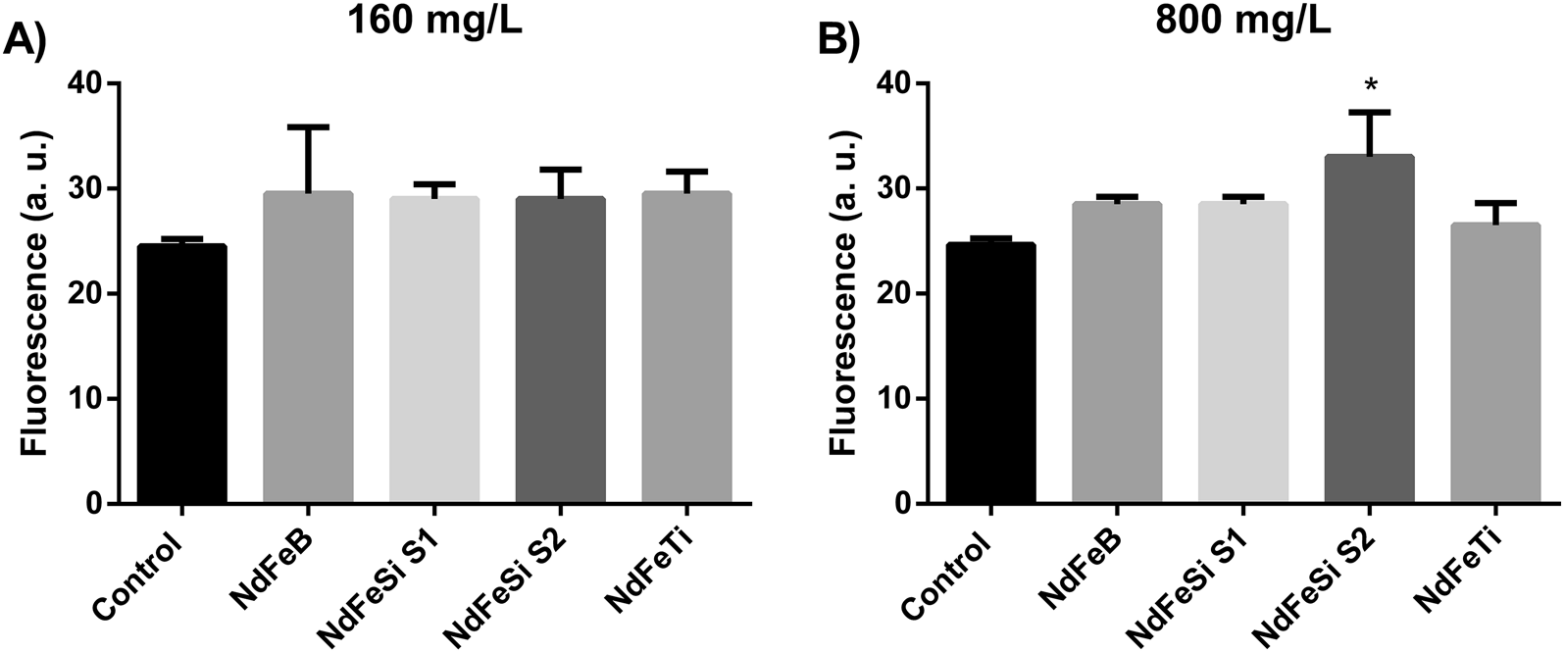
ROS induction analysis of *S. cerevisiae* cells exposed to different alloys during 2 h at two different concentrations (160 and 800 mg/L). Results are expressed as arbitrary fluorescence values. Data represent the mean of 2 biological replicates (± standard deviation, SD). Differences were established using a One-way ANOVA followed by Dunnett post hoc test to compare every mean with the control condition, and considered significant at *P* ≤ 0.05. *P ≤ 0.05.

The toxicological potential of the alloys suspensions in *S. cerevisiae* was also determined investigating their ability to induce oxidative stress. As in the viability experiments, concentrations of 160 and 800 mg/L were used to expose yeast cells for 2 h (Fig. 6). In this case, only a statistically significant increase in the ROS levels could be observed in the yeast cells exposed to 800 mg/L of NdFeSi S2 sample. The observed results, considering the high concentrations used in the exposure experiment, indicate a low ability of the different alloys to induce oxidative stress in *S. cerevisiae.*

### Toxicity determination of the alloys leachates applying the *Vibrio fischeri* bioluminescence inhibition assay

**Figure 7:**
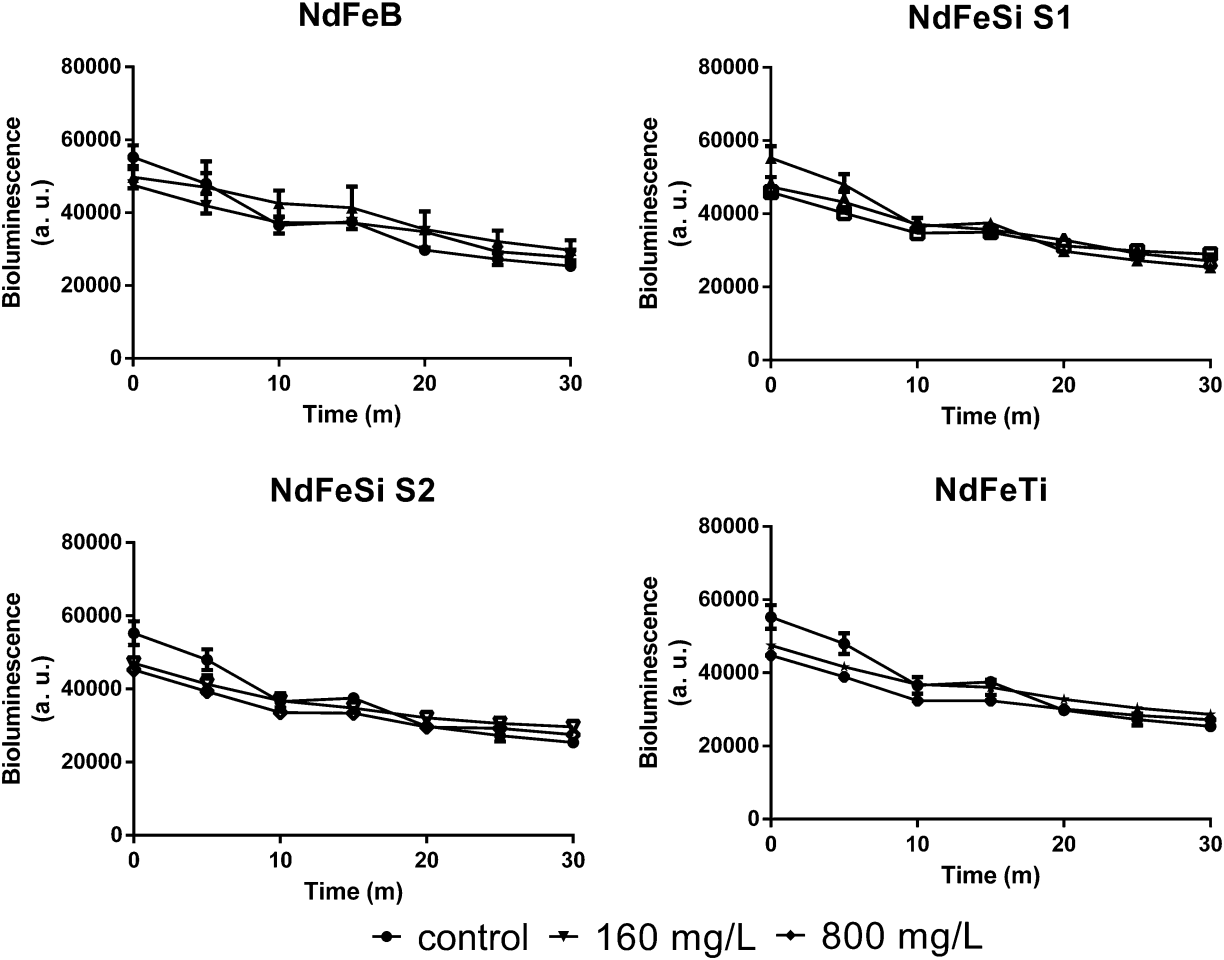
Evolution of *V. fischeri* bioluminescence intensity during the 30 min-exposure to alloys leachates. Each point represent the mean of 3 biological replicates (± standard deviation, SD).

Due to their optimal sensitivity and reliability, tests applying *V. fischeri* as model organism are frequently used for the detection and ecotoxicological screening of toxic substances in a variety of substrates, including soils and water^27^, as well as for the study of the safety of different elements such as REs and metals^28^. To determine whether the ion levels detected in the alloy leachates were above the safety limits, *V. fischeri* was exposed to them at different concentrations with the aim to study their effect on the natural bioluminescence of this microorganism. As shown in Fig. 7 and Table 2, none of the leachates led to an inhibition of the light intensity at the concentrations tested. Figure 7 displays the evolution of the bioluminescence of *V. fischeri* during a 30 min-incubation period in the presence of leachates equivalent to concentrations of 160 and 800 mg/L of the alloys. A significant drop in the initial bioluminescence peak value was observed in all the samples incubated with the leachates. However, from this point on, the intensity of the light barely decreased, presenting all samples similar bioluminescence decrease patterns along time. The bioluminescence inhibition percentages after 5, 15 and 30 min exposure are showed in Table 3. Negative values were obtained for all the samples at all the time points tested, which indicates that the drop in the light intensity during the incubation was lower than the natural attenuation of the microorganism even in the early stages of the incubation.

**Table 2:** Bioluminescence inhibition of *V. fischeri* cells exposed to different concentrations of alloy leachates at 5, 15 and 30 min (percentage of control).

Sample	% of bioluminescence inhibition		
	5 min	15 min	30 min
NdFeB 160 mg/L	− 8.35 ± 9.77	− 22.08 ± 9.63	− 29.91 ± 4.07
NdFeB 800 mg/L	− 1.47 ± 3.63	− 15.35 ± 4.30	− 27.52 ± 11.68
NdFeSi S1 160 mg/L	− 0.77 ± 3.94	− 12.23 ± 4.04	− 37.59 ± 7.17
NdFeSi S1 800 mg/L	− 4.88 ± 4.37	− 11.07 ± 6.24	− 24.85 ± 6.39
NdFeSi S2 160 mg/L	− 1.01 ± 3.97	− 8.98 ± 3.67	− 37.45 ± 2.91
NdFeSi S2 800 mg/L	− 0.12 ± 3.06	− 8.67 ± 5.05	− 32.51 ± 4.17
NdFeTi 160 mg/L	− 0.82 ± 2.16	− 11.73 ± 6.48	− 31.33 ± 4.98
NdFeTi 800 mg/L	− 0.14 ± 2.64	− 6.55 ± 2.76	− 32.35 ± 5.79

**Table 3:** Samples, alloy composition and crystal structure of the materials analysed in this study.

Sample name	Alloy composition	Crystal structure
NdFeB	Nd_2_Fe_14_B	P4_2_/mnm, space group 136
NdFeSi S1	(Nd_0.2_Zr_0.7_Y_0.1_)Fe_10_Si_2_	I4/mmm, space group 139 (1:12)
NdFeSi S2	(Nd_0.2_Zr_0.7_Y_0.1_)Fe_10_Si_2_	I4/mmm, space group 139 (1:12)
NdFeTi	(Nd_0.75_Pr_0.25_)_1.15_Fe_11_Ti	I4/mmm space group 139 (1:12)

## Discussion

The rapid growth of industries where metallic powders are applied, together with the risks that they may represent for health and the environment^29^, make the availability of data concerning the potential toxicity of these materials a priority issue. The application of additive manufacturing technologies in the permanent magnet field involves the use of metal powders, for example, for the production of 3D-printed magnets^30^. Hence, it is important to know the specific hazards associated to their manipulation and to the wastes generated after their use, considering not only the potential impact of the small metal particulates, but also that of their corrosion products, which can be a source of contamination through the release of metallic ions to the environment, where they may pose substantial negative consequences^31^. In this study, the toxicological potential of three RE lean alloys with the same crystal structure (two types with the composition (Nd_0.2_Zr_0.7_Y_0.1_)Fe_10_Si_2_ and one type with the composition (Nd_0.75_Pr_0.25_)_1.15_Fe_11_Ti), developed as alternatives for current NdFeB permanent magnets, was evaluated applying different in vitro assays and using model organisms representative of human and environmental exposures.

Firstly, the powders and leachates under study were characterized through different techniques. Thus, the surface elemental composition of the alloys was semi-quantitatively analysed through SEM–EDX, confirming that the reference NdFeB sample presented the highest percentage of Nd in its composition, while both NdFeSi samples had the lowest content of this element. In the case of the NdFeTi sample, and in spite of the fact that the reduction of this element was not as significant as in the others, it also represents a relevant decrease in the final Nd content. In addition, due to the fact that morphology and size of the particles are factors that could influence the toxicity of the powder alloys, their appearance was analysed and observed by SEM. It was confirmed that all the samples used in this work consisted in a heterogeneous population of particles presenting irregular morphologies (mainly polygonal shapes) and a variety of sizes in the micro and nanometric scales, with a minor difference in the biggest particles size, which were found to be slightly larger in the RE-lean alloy powders, probably due to the different methodologies applied for their generation (jet milling was used to obtain the NdFeB powders, while RE-lean alloy powders were produced after smashing magnet pieces with a mortar). Regarding the leachates analysis, different concentrations of Fe, Nd or Si, as well as Pr or Y, which were applied during the fabrication of some of the alloys, were detected by ICP-MS in the samples obtained directly from the alloys used in the experiments. Unexpectedly, the concentration of Nd in NdFeB leachates was very low compared to that quantified in the RE-lean alloys, which present lower percentage of Nd in their composition. Since the morphology of the particulates is similar in all the samples and none of the alloys were coated, these variations, as well as those observed between both NdFeSi samples, could be attributed to the different susceptibility to metal leaching of the elements applied during the alloys manufacturing.

Due to the widespread distribution of metals, their impact in the biological systems is a topic of critical interest and many research works have addressed this issue. In the present work, to evaluate the toxicological potential of the direct interaction of the RE-lean alloy powders, the human cell line A549 and the yeast *S. cerevisiae* were used. Moreover, the effect of the alloy leachates was studied in both organisms and in the luminescent bacterium *V. fischeri*. Our results showed that only the direct interaction of high concentrations of the particulates were able to cause harmful effects. More specifically, statistically significant levels of oxidative stress were observed in human cell lines after being exposed to 160 mg/L of all the alloy powders, while the viability of *S. cerevisiae* was compromised when exposed to NdFeB and NdFeSi powders at 800 mg/L. The number of available studies assessing the toxicity of RE-permanent magnets is scarce, with most of them mainly focused on the analysis of Sm and Nd alloys, two of the most common materials applied in dentistry. In the specific case of Nd magnets, several research works have been performed analysing the safety of magnet pieces in the mm scale using mammalian cell lines, and reaching in some cases different conclusions regarding their potential cytotoxicity^21–23^. Concerning the biological impact of permanent magnet particulates, Périgo *et al.* showed that MRC-5 cells exposed to NdFeB nanoparticles coated with oleic acid at concentrations up to 100 µg/mL presented a viability ≥ 80%^32^. Moreover, in a recent study where we described the potential toxicity of MnAl based magnets powders, the safety of a commercial NdFeB magnet powder, used as reference, was evaluated too in the A549 cell line and *S. cerevisiae*, showing similar effects to those observed on the NdFeB magnet studied in the present work^24^. The safety of metal micro and nano-sized particles in eukaryotic cellular models has been addressed by different studies. For instance, Armstead *et al.* reported that both nano- and micro-CoCrMo can induce toxicity, showing that the effects on cell viability and oxidative stress were specific to dose, exposure time and cell type^33^. For its part, Li *et al.* described the toxicity of different elemental metal powders in osteoblast-like SaOS2 cells^34^. These authors showed that in some cases the elemental metals exhibited different degrees of cytotoxicity depending on if the materials were in bulk or powder forms, the latter presenting higher toxicity. These authors associated these differences with the higher ability of powdered metals to release ions to the medium, resulting, therefore, more cytotoxic. By the same token, Palombella *et al.* carried out a comparative multidisciplinary study based on the size effect of zero-valent iron, cobalt, and nickel microparticles and nanoparticles using human adipose stem cells (hASCs) as model organism, and evaluating different parameters such as cytotoxicity, morphology, cellular uptake, and gene expression^35^. These authors observed that while nanoparticles were mainly internalized by endocytosis and could persist in the vesicles without any apparent cell damage, microparticles were not internalized. Thus, these authors suggested that the negative effects observed in hASCs could be explained by the release of ions in the culture medium, or to the reduced oxygen and nutrient exchange efficiency that the presence of microparticles around the cells could cause.

Metal ions’ toxicity in *S. cerevisiae* has been reported to be similar to that observed in animal cells^36^. Mechanistic toxicity analyses of different metallic elements in yeast indicate a fast and general activation of metal-specific oxidative defence and protein degradation processes^25^. This fast response allows a rapid adaptation to acute metal exposure via effective establishment of metal defences at proteomic level, resulting in a relatively fast reduction of the detoxification pathways activity, which could explain why the observed ROS levels in the metal alloys exposure conditions were similar to those shown by the non-exposed yeast cells.

Since all the alloys leachates used in the present study showed to be safe for the different organisms, it is more feasible to attribute the oxidative stress observed in A549 cells, as well as the decrease in the viability of *S. cerevisiae*, to the direct interaction of the micro and nano-sized particles of each sample with the selected cellular models. Thus, these observed effects are probably consequence of the combined action of the microparticles outside of the organisms, together with the effects of the nanoparticles, that could be internalized and cause cellular stress by different mechanisms, as it was already described for different metal oxide nanoparticles in A549 cells^37^.

Both essential (Cu, Fe, Mn, Zn…) and not essential (Al, As, Cd, Pb, Hg…) metal ions can cause toxicity. Essential metal toxicity is mainly due to their excessive accumulation in the organism, while the harmful effects induced by not essential metals are associated to both their accumulation and to the disturbance of essential metals’ function^15^. Szivák *et al*. reported that environmental low concentrations of some metals such as Fe, Pb or Cd are enough to produce an increase in the intracellular levels of ROS in cells of the freshwater alga *Chlamydomonas reinhardtii*^38^. In this regard, it is also important to remark that, in the environment, organisms are generally exposed to mixtures of metals so, in spite of being under toxic levels, their combination can result in adverse consequences^17^. This effect is usually underestimated since many research works are focused in the study of the organisms´ responses to these ions individually. In our assays, the organisms were exposed to alloy leachates equivalent to the concentrations used in the direct contact experiments, by which they contain mixtures of ions. This constitutes one of the strengths of the present work, since, as explained above, it is representative of more realistic conditions. None of the leachates showed to be toxic at the different concentrations tested, confirming that the metal ions mixture presented in the samples do not pose a significant challenge for any of the organisms applied. These results are concordant with those obtained in our recent work, focused on the evaluation of the toxicity of MnAl based permanent magnets, where the leachates of NdFeB commercial powders were also evaluated in the same organisms and conditions^24^.

## Conclusions

The safety of three Nd-lean alloys with the same crystal structure and designed to be applied in the manufacturing of permanent magnets was evaluated in this work. The selected alloys included two NdFeSi and one NdFeTi alloys, and a commercial NdFeB sample, which was used as reference.

The potential toxicity of the alloys in powder form and their associated leachates was analysed using three model organisms which can be considered representative of human (A549 cells) and environmental exposures (*S. cerevisiae* and *V. fischeri*) and applying different in vitro assays. In spite of the fact that some works have suggested that metal particles toxicity is attributable to their ability to release metal ions to the surrounding environment, our results showed that only the direct interaction of the alloy particulates with the organisms have resulted in pernicious effects, being the observed effects comparable to those caused by the commercial powders. Thus, oxidative stress was statistically significant in A549 cells exposed to 160 mg/L of the alloys, while in *S. cerevisiae*, yeast cells exposed to both NdFeSi samples showed a reduction in their viability at 800 mg/L, being statistically significant in the case of NdFeSi S2.

In summary, the present work constitutes a preliminary analysis of the biological impact of three specific RE-lean alloys, indicating that the particles of these specific materials could pose a toxicological risk due to their direct interaction with the organisms, while the released ions seems not to be involved in their toxicity. However, more research is needed to characterize in depth the effects of these materials, as well as their behaviour in more realistic scenarios.

## Methods

### Alloys and sample preparation

The materials used in this study are specified in Table 3.

NdFeSi S1 ((Nd_0.2_Zr_0.7_Y_0.1_)Fe_10_Si_2_ strip-cast flakes) were produced by the strip-casting technique. 15 kg of pure elements (purity higher 99.5%) were melded inductively in an alumina crucible under inert atmosphere. The melt was poured at 1350 °C via a tundish onto a water-cooled copper wheel. The surface wheel speed of the quenching wheel was set to 1.8 m/s to avoid formation of α-Fe. The flakes were crushed after the quenching to flakes with a diameter of approximately 20 mm and a thickness of 320 µm.

NdFeSi S2 ((Nd_0.2_Zr_0.7_Y_0.1_)Fe_10_Si_2_ melt-spun ribbons) were synthesized by the melt-spinning technique. 50 g of the strip-cast flakes were used as input material. During melt-spinning, the flakes were melted inductively in a quartz glass crucible in argon atmosphere. At 1355 °C the melt was casted trough a rectangular nozzle (0.6 × 7 mm^2^) by an Ar overpressure of 350 mbar and subsequently quenched onto a water-cooled copper wheel. The surface wheel speed was set to 10 m/s resulting in flakes with a finer microstructure and smaller grains compared to the strip-cast flakes of NdFeSi S1.

NdFeTi ((Nd_0.75_Pr_0.25_)_1.15_Fe_11_Ti ribbons) was produced like the NdFeSi S1 sample, but using different equipment. Thus, the strip-casting technique was applied, i.e., induction melting followed by casting on a water-cooled rotative copper wheel with an strip-casting furnace from ULVAC. The linear speed of the wheel was set to 1.4 m/s.

As reference sample, commercial Nd_2_Fe_14_B alloy powders provided by ARELEC Magnets and Magnetic Systems were used. This sample was produced by strip casting and jet milling, and it was named in this work as NdFeB.

With the aim to obtain a uniform powder, NdFeSi S1, NdFeSi S2, and NdFeTi samples were smashed in a mortar. The obtained powder was used to prepare the stock solutions for the experiments. In the case of the reference sample, the powder provided by ARELEC was used directly to prepare the stock solutions.

Stocks of the powder alloys resuspended in water at 10 g/L were prepared to perform the direct contact experiments. In order to obtain the alloy leachates, powders were left in water at 10 g/L during 3 months at 4 °C. After this incubation, the samples were centrifuged and the supernatants with the leachates were recovered and subsequently filtered through 0.22 polyethersulfone membranes. Before performing direct contact tests, the alloy powders were treated to obtain a homogenized solution as described below: samples were vortexed at full speed for 1 min and then submitted to ultrasonication for 20 min at low power intensity. Finally, before preparing the different concentrations used in the experiments, an additional vortex step was performed.

### Analysis of the surface composition and morphology of the alloys

The surface composition of the alloys used in this work was semi-quantitatively analysed by SEM–EDX (Scanning Electron Microscopy–Energy Dispersive X-ray spectroscopy) in the University of Burgos using a JEOL JSM-6460LV microscope equipped with a X-Max^N^ energy dispersive detector. To perform the element identification and quantification, at least 3 different areas were selected and analysed in each material.

The morphology and the size of the particles that are part of the different alloys powders were analysed by Scanning Electron Microscopy. A small quantity of each sample was directly examined using JEOL JSM-6460LV at the Microscopy Facility of the University of Burgos.

### ICP-MS analysis of the alloys leachates

Water samples containing the leachates from alloys aqueous suspensions (10 g/L), obtained after incubate powders at 4 °C for three months, where analysed by inductively coupled plasma mass spectrometry (ICP-MS) using an Agilent 8900 ICP-QQQ instrument. For data acquisition, 5 replicates were used.

### Model organisms and culture conditions

A549 lung cancer cell line was purchased from Sigma. It was cultured in commercial Dulbecco’s Modified Eagle’s Medium (DMEM) supplemented with 10% (v/v) fetal bovine serum (FBS) and 100 U/mL penicillin and 100 mg/L streptomycin, and incubated under standard conditions in 37 °C humidified 5% CO_2_ atmosphere.

The BY4741 strain of *S. cerevisiae* was grown and maintained in standard YPD medium (1% yeast extract, 1% yeast bacto-peptone, 2% glucose), while cell cultures in liquid media were done on a rotary shaker at 185 rpm at 30 °C.

The Gram negative bacterium *V. fischeri* NRRL B-11177 was purchased from the German Collection of Microorganisms and Cell Cultures GmbH (DSMZ), and maintained at room temperature in Marine Broth or Agar 2216.

### Viability test in A549 cells: neutral red uptake assay

The neutral red uptake assay was used to determine the viability of A549 cells after being exposed to the magnetic alloys following a previously described protocol^24^. Briefly, cells were seeded in 96 well plates (3 × 10^4^ cells per well) and, 24 h after seeding, cells were incubated for 24 h with different concentrations of the alloys (6.4, 32, and 160 mg/L) resuspended in fresh treatment medium, which consisted in DMEM with 1% FBS and without antibiotic. As controls, cells incubated with treatment medium alone (live cells control) were included in the experiments. After the incubation, wells were washed with DPBS, and 100 µL of a neutral red solution (stock of neutral red at 4 mg/mL diluted 1:100 in treatment medium) were added to each well for 2.5 h. After this time, the solution was discarded, cells were washed once with DPBS and subsequently fixed with formaldehyde 4% for 2 min. Cells were then washed again with DPBS and a dye release solution (150 µL) consisting of 50% ethanol 96°, 49% distilled H_2_O and 1% acetic acid was added to each well. Plates were shaken for 10 min, and 100 μL of each well were transferred to an opaque 96-well plate. Fluorescence was measured using a microplate reader (BioTek Synergy HT, excitation wavelength, 530/25; emission wavelength 645/40). Results were expressed as percentage of control (fluorescence of cells in absence of alloys), and each assay included three biological replicates. To test the toxicity of the alloy leachates, cells were exposed to different dilutions of the stock samples during 24 h, and the viability was analysed using the above-explained protocol.

### Oxidative stress assay in A549 cells

A549 cells were seeded in 96 well plates at 3 × 10^4^ cells per well, washed once with Hank’s Balanced Salt Solution (HBSS) without phenol red, and incubated with a solution of DCFH-DA (2ʹ,7ʹ-Dichlorofluorescin Diacetate) in HBSS (50 µM) for 30 min at 37 °C in the dark. After the incubation, cells were washed once with HBSS, and exposed to different concentrations of alloys (6.4, 32, and 160 mg/L) resuspended in HBSS. Cells incubated with HBSS alone were used as control. Fluorescence was measured after 60 min of incubation using a microplate reader (BioTek Synergy HT, excitation wavelength, 485/20; emission wavelength 528/20). Each assay included three biological replicates. The oxidative stress induced by different dilutions of the leachates was analysed applying the above-explained protocol.

### *S. cerevisiae* viability assay: colony forming units (CFUs) determination

Yeast cells in exponential growth phase (OD_600_ = 1) were exposed to the different alloys at 160 and 800 mg/L in 1 mL cultures performed in 24 well plates. Yeast colony forming units were determined for two exposure times (2 and 24 h), cells were inoculated on solid YPD medium (6% agar) and incubated at 30 °C. The toxicity of the leachates equivalent to 800 mg/L of the alloys was tested following the above-explained protocol.

### Oxidative stress assay in *S. cerevisisae*

Intracellular levels of ROS were determined using the reagent CM-H2DCFDA (5-(and-6)-chloromethyl-2′,7′-dichlorodihydrofluorescein diacetate, acetyl ester), following a protocol previously described^39^. Briefly, *S. cerevisiae* cells growing in exponential phase were pelleted, washed and incubated with CM-H2DCFDA (7 µM) in DPBS for 60 min at 30 °C and 185 rpm. Subsequently, cells were washed again, resuspended in YPD and exposed to the different alloys (160 and 800 mg/L) for 2 h. Then, yeast cells were washed two times with DPBS, incubated 2 min in a solution containing Lithium Acetate 2 M, washed and incubated again for 2 min in a solution containing SDS (0.01%) and chloroform (0.4%). Cells were finally pelleted and the supernatant transferred to a black opaque 96 micro-well plate, where the fluorescence was measured (excitation wavelength, 485/20; emission wavelength 528/20) using a microplate reader (Synergy-HT, BioTek).

### Bioluminescence inhibition assay in *V. fischeri*

The impact of the alloy leachates on the luminescence produced by *V. fischeri* was studied applying the bioluminescence inhibition assay. 5 mL of Marine Broth 2216 were inoculated with one luminescent colony and cultured for 48 h. After the incubation, the bacterial suspension was centrifuged, resuspended in 5 mL of NaCl 2% (w/v), and pre-incubated at 10 °C for 30 min before starting the experiment. 90 µL of leachates resuspended in NaCl 2% solution (leachates equivalent to concentrations of 160 and 800 mg/L of the alloys) were added to each well in a 96 well opaque microplates. Bacteria cultured in 2% NaCl were used as control for the natural light attenuation of this microorganism. 10 µL of the bacterial suspension were added into each well of the microplates, and the luminescence was immediately measured to obtain the initial peak value using a microplate reader BioTek Synergy HT. The microplate was then incubated in a Thermomixer at 800 rpm and 15 °C, and the luminescence of each sample was recorded in 5 min intervals throughout 30 min in a microplate reader (Synergy-HT, BioTek). The inhibition of luminescence (percentage of control) was calculated at different time points using the values obtained at 5, 15 and 30 min (M-value) using the following formula, adapted from Jarque *et al.*^40^:

$$INH\%=100-\frac{M}{CF\times peak } \times 100$$

where CF is a correction factor (the *M*/peak ratio in negative controls) reflecting natural attenuation of bacterial luminescence after 5, 15 or 30 min of incubation.

### Statistical analysis

Data are presented as means ± SD. Statistical analysis of the viability and oxidative stress assays in both A549 cell lines and *S. cerevisiae* was performed by the one-way analysis of variance (ANOVA), followed by Dunnett post hoc test to stablish comparisons between every mean and the control. Statistical tests were carried out using Prism 6.0 (GraphPad Prism, GraphPad Software, Inc.), considering the differences significant at *P* ≤ 0.05.
